# In Transected Nerves, Distal Repair Schwann Cells at the Injury Site Direct and Accelerate Axonal Regrowth

**DOI:** 10.1002/glia.70214

**Published:** 2026-08-07

**Authors:** Daniel E. Lysko, Annika R. Johnson, William S. Talbot

**Affiliations:** ^1^ Department of Developmental Biology Stanford University Stanford California USA

## Abstract

In vertebrate peripheral nerves, damaged axons can regrow after injury, but the outcomes of regeneration are variable and often incomplete. Schwann cells in injured nerves are important for repair, but their actions at different positions and stages of nerve repair are not well understood. We have investigated the roles of Schwann cells in a larval zebrafish nerve injury model, in which nerves are visible in living animals during development, the initial injury response, and regrowth of transected axons. After mechanical injury, distal Schwann cells adopted a repair phenotype characterized by changes in gene expression, elongation, and ability to guide axons across the injury site. In contrast, proximal Schwann cells associated with axons that regrew along aberrant paths at the injury site. In *erbb2* mutants with greatly decreased Schwann cells, developmental axon growth was normal, but axonal regrowth after transection was greatly slowed and often misdirected. By examining animals with nerves partially populated by Schwann cells, we found that axons could regrow through regions devoid of Schwann cells, provided that at least one distal Schwann cell was at the injury site. Timelapse imaging revealed that distal Schwann cells contacted axons, which then regrew along the correct path. In *irf8* mutants with greatly decreased macrophages, debris from transected axons was cleared on schedule, and axonal regrowth was normal. Our studies demonstrate that Schwann cells immediately distal to the injury site have a unique and essential role in axonal regrowth.

## Graphical abstract

- After nerve transection in larval zebrafish, proximal and distal Schwann cells have distinct functions at the injury site.
- Distal repair Schwann cells promote axonal regrowth.
- Axonal regrowth is normal in mutants with only a few macrophages.

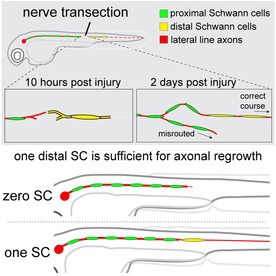

## 1 Introduction

Elucidating the mechanisms that control the repair of the nervous system after injury remains a great challenge (Jessen and Mirsky 2016). One challenge is the nature of injury, which may vary widely in region, scope, and life stage. For example, repair of a nerve in an adult may involve regrowth of axons over distances much greater than those in the embryo, and at stages when the developmental guidance cues are no longer operative. Furthermore, the debris and inflammation generated by injury may impede regrowth and repair (Rotshenker 2011; Brosius Lutz et al. 2017; Reed et al. 2020). On the other hand, injury occurs in the context of a pre‐existing, functional nervous system, which may provide a template for repair.

Schwann cells in peripheral nerves adapt to injury of their associated axons and promote axonal regrowth, providing a compelling example of cellular redeployment after injury (Arthur‐Farraj et al. 2012). After a nerve is transected, the distal regions of severed axons degenerate (Waller 1851; Beirowski et al. 2005). The denervated Schwann cells greatly alter their functions to adopt a distinct cell state that promotes repair (Arthur‐Farraj et al. 2012, 2017). During development and homeostasis, Schwann cells make and maintain myelin, but in stark contrast, repair cells degrade myelin and engulf axonal debris, thereby clearing the distal nerve segment to allow axonal regrowth (Jessen and Mirsky 2016; Jessen and Arthur‐Farraj 2019). Repair cells also attract macrophages, which contribute to debris clearance after injury (Stoll et al. 1989; Perry et al. 1995; Dailey et al. 1998; Villegas et al. 2012; Morales and Allende 2019). In mammals, repair Schwann cells greatly increase their length, forming characteristic structures called Büngner bands that provide a substrate for axons regrowing through the distal nerve segment (Gomez‐Sanchez et al. 2017). Thus, injury reverses the relationship between Schwann cells and axons: axons guide Schwann cell migration during development but are apparently guided by repair Schwann cells during regrowth (Gilmour et al. 2002; Ceci et al. 2014; Jessen and Mirsky 2016).

Many studies demonstrate that Schwann cells are essential for nerve regeneration after injury (Jessen and Mirsky 2016; Bosch‐Queralt et al. 2023). In zebrafish mutants lacking Schwann cells, motor and sensory axons are relatively normal during initial outgrowth, but axonal regrowth after injury is delayed, misrouted, and incomplete (Gilmour et al. 2002; Lyons et al. 2005; Raphael et al. 2010; Perlin et al. 2011; Villegas et al. 2012; Ceci et al. 2014; Rosenberg et al. 2014; Xiao et al. 2015; Walker et al. 2023). In mammals, c‐Jun is essential for Schwann cells to convert to the repair state, and conditional c‐Jun mutants have diminished myelin clearance, disrupted axonal regrowth, and impaired functional recovery (Arthur‐Farraj et al. 2012; Ruff et al. 2012). Although repair Schwann cells play key roles following injury, important questions remain about the distinct functions of Schwann cells in different positions along the injured nerve, including the distal and proximal nerve segments and the injury site itself (Graciarena et al. 2014; Xiao et al. 2015; Clements et al. 2017). Furthermore, the requirement of macrophages for debris clearance and axon regrowth remains to be clarified: macrophages can phagocytose debris after injury, but robust axonal regrowth can still occur in some animals in which macrophages have been reduced or eliminated (Lunn et al. 1989; Stoll et al. 1989; Perry et al. 1995; Villegas et al. 2012). Zebrafish mutants with only a few macrophages (Shiau et al. 2015) present an opportunity to test the requirement of macrophages specifically for sensory axon regrowth after transection.

In the present study we have developed a mechanical nerve injury model in the larval zebrafish, which is genetically tractable and allows in vivo imaging of nerves before, during, and after nerve injury and repair. We focus on the posterior lateral line nerve (PLLN), which transmits sensory input from neuromasts sensing water flow to the hindbrain (Liao 2010). During the initial phase of posterior lateral line development, the lateral line primordium migrates in the skin along a roughly linear path from the ganglion near the ear to the tip of the tail, starting at about 22 h post fertilization (hpf) and concluding at about 40 hpf (Figure 1B,C) (Kimmel et al. 1995; Nechiporuk and Knaut 2026). The migrating primordium precedes and directs the outgrowing PLLN axons (Metcalfe 1985; Gilmour et al. 2004; Grant et al. 2005), which in turn direct the migration of accompanying Schwann cells (Gilmour et al. 2002; Lyons et al. 2005; Perlin et al. 2011). Schwann cells are required to shift the location of the axons from a superficial position in the skin to a deeper position beneath the skin basement membrane (Raphael et al. 2010). By 3 days post fertilization (dpf), active neuromasts are innervated, and several myelinated axons are evident in the PLLN (Sarrazin et al. 2010; Raphael et al. 2011; Nechiporuk and Knaut 2026).

**FIGURE 1:**
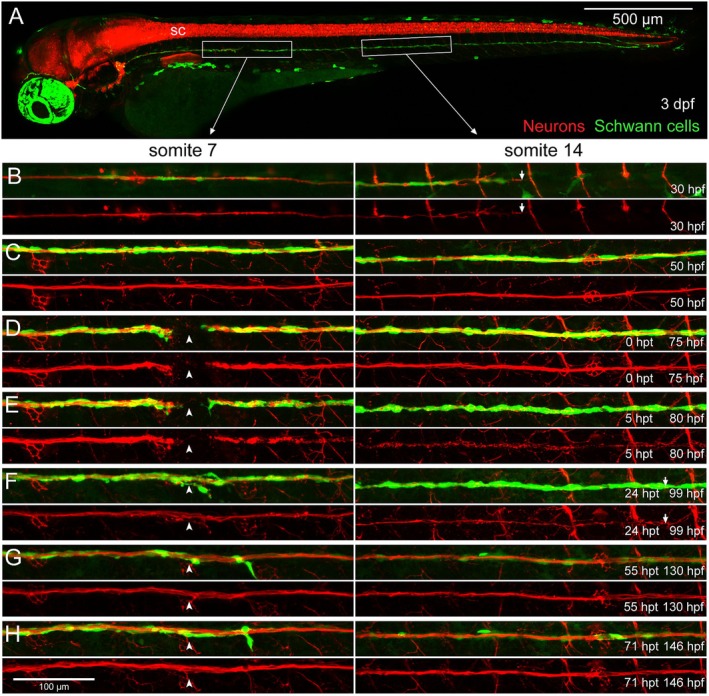
Time course of posterior lateral line development and recovery after transection in wildtype larvae. (A) Wildtype larva with transgenic markers of Schwann cells (*foxd3:GFP*, green) and neurons (*NBT:dsRed*, red) a few hours after mechanical injury of the PLLN at the region of somite 7 at 3 dpf. White boxes denote regions shown at higher magnification in subsequent panels. The spinal cord (sc) and other deep structures appeared intact. (B–H) Time course of images showing development and regrowth of PLLN after mechanical injury (arrowheads mark injury site). (C–H) Images of the same fish at the indicated stages. Different animals are shown in (A) and (B). The tips of growing axons (arrows) are noted during development (B, 30 hpf) and regrowth (F, 24 hpt).

Complementing prior models using laser and electroablation (Rosenberg et al. 2012; Villegas et al. 2012; Moya‐Díaz et al. 2014; Xiao et al. 2015), our mechanical injury model is rapid, reproducible, and accessible. We find that Schwann cells distal to the injury site display patterns of gene expression and other characteristics of repair Schwann cells previously defined in mammals. Repair Schwann cells elongate and extend processes that contact axons regrowing across the injury site (Stoll and Müller 1999; Ceci et al. 2014; Xiao et al. 2015; Gomez‐Sanchez et al. 2017; Walker et al. 2023). We found that axonal regrowth is impaired in the absence of any distal Schwann cells, but that extensive regrowth can occur when only a single distal Schwann cell is present. Finally, we found that axonal regrowth was normal in mutants with greatly decreased macrophages. Our studies demonstrate that distal repair Schwann cells at the injury site have unique and essential functions in axonal regrowth.

## 2 Methods

### 2.1 Zebrafish Lines and Maintenance

All zebrafish experiments were conducted using protocols approved by the Stanford University institutional animal care and use committee and conforming to appropriate city, state, and national regulations. Embryos and larvae were anesthetized with 0.016% Tricaine (Syncaine, Syndel) prior to experimental procedures. Embryos and larvae were treated with 1‐phenyl 2‐thiourea (PTU, 0.003%, Sigma) to inhibit pigmentation for microscopy. Embryos and larvae were analyzed up to 6 dpf. The *erbb2* ^st61^ and *irf8* ^st96^ mutant lines have been previously described (Lyons et al. 2005; Shiau et al. 2015).

### 2.2 Nerve Transection and Axonal Regrowth Analysis

Larvae were anesthetized with Tricaine and mounted on their side on a coverslip in 0.9% agarose (UltraPure LMP Agarose, Invitrogen). The left PLLN was transected at approximately the seventh somite (muscle segment along the body) (±1 somite) with an insect pin (stainless steel Minutien pins, 0.02 mm tip, 0.2 mm rod, FST) (diagram in Figure S3A). Immediately after injury, the larvae were imaged to confirm full transection of the nerve using a Zeiss LSM700 confocal microscope. Axons were visualized using the *Tg(NBT:dsRed)* line (Peri and Nüsslein‐Volhard 2008). During this initial injury screening, we excluded any larvae that did not have fully transected nerves and those that had excessive injuries, such as injury to the spinal cord or yolk. Axonal regrowth was quantified by counting somites of regrowth (the number of somites through which regrowing axons projected from the injury site) (diagram in Figure S3A). Within a trial, axons in experimental and control animals were measured within a short time window to minimize variation due to ongoing regrowth during the analysis. Schwann cells were visualized using the Tg(z*Foxd3:GFP*(17)) line (Gilmour et al. 2002). Remyelination was visualized with a *Tg(mbp:GFP‐CAAX)* line (Almeida et al. 2011). Macrophages were visualized using the *Tg(mpeg1:GFP)* line (Ellett et al. 2011).

### 2.3 Schwann Cell Length Measurements

It is challenging to measure the length of individual Schwann cells using the *foxd3:GFP* transgene after the PLLN axons have completed outgrowth because individual Schwann cells cannot be readily distinguished among the many overlapping cells in the wildtype nerve (Gilmour et al. 2002; Ceci et al. 2014). During PLLN outgrowth, Schwann cells at the growing tips of the axons are less dense, and close observation utilizing confocal microscopy Z‐stacks allowed us to measure the length of individual leading Schwann cells in some of the animals we examined. At 30 hpf, the leading axon tip and Schwann cell are near somite 15, and we found that transection of the nerve at this stage at the position of somite 7 removes the axon from the leading Schwann cell. The leading Schwann cell frequently migrates a bit further as the axon fragments, becoming isolated and making post‐injury length measurement easier (Figure 2E–H). In animals with nerves partially covered with Schwann cells, the lengths of distal Schwann cells were also measured, as individual cells could be distinguished on the distal side of the injury site (Figure 7).

**FIGURE 2:**
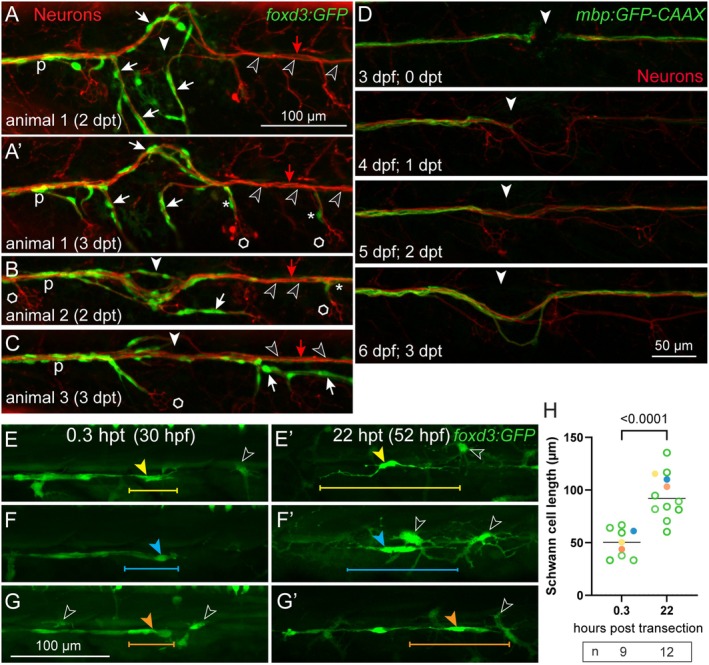
Distal Schwann cells display characteristics of repair Schwann cells. (A–C) Three different examples of axons regrowing at the site of mechanical injury (white arrowhead). The anterior (proximal) side of the nerve is to the left in all images. Although all Schwann cells express the *foxd3:GFP* transgene (green), proximal Schwann cells (p) continue to highly express *foxd3:GFP*, while by 2 dpt, distal Schwann cells (black arrowheads) have weak expression (5‐fold less than the corresponding proximal segment). Axons regrowing along the correct path (red arrow) associated with distal Schwann cells that weakly express the reporter, whereas more strongly expressing proximal Schwann cells associated with misrouted axons (white arrows, 3‐fold more GFP signal than Schwann cells on axons that regrew along the correct path). (A′, B) By 2–3 dpt, one to two GFP‐expressing Schwann cells (*) associated with axons projecting from the main nerve trunk to innervate neuromasts (hexagons). Neurons are labeled with the *NBT:dsRed* transgene (red). (D) Time course of expression of *mbp:GFP‐CAAX* myelin reporter (green) and neuronal reporter *NBT:dsRed* (red) after mechanical injury and nerve regrowth. By 1 dpt, axons had regrown through the injury site (arrowhead), and myelin reporter expression was greatly reduced in the distal segment (right side of the image, 5‐fold less GFP signal than proximal segment). By 3 dpt, myelin reporter expression had recovered, so that the distal segment had 2.5‐fold less GFP than the proximal segment. (E–G) Schwann cell morphology immediately after injury (30 hpf, 0.3 h after transection). The injury site (not pictured) was to the left of the region shown, that is, proximal to the migrating cells that are depicted. Colored arrowheads note the leading Schwann cell at the tip of the growing axons in three distinct animals. Brackets annotate the length of the leading Schwann cell. Pigment cells (open arrowheads), which exhibit characteristic stellate morphology, also express the *foxd3:GFP* transgene at this time point. (E′–G′) 22 h post transection (hpt), the same Schwann cells have become isolated after axonal loss and greatly increased in length, extending fine processes and stretching along the path of the PLLN. Brackets annotate the length of these Schwann cells. (H) Schwann cell length is significantly increased 22 hpt. The length of Schwann cells in E–G′ denoted as filled circles; graphed with additional non‐pictured Schwann cells (open circles). *n* = one leading Schwann cell in a distinct animal, one experiment. Within the cohort of animals, only Schwann cells that could be discriminated from neighboring Schwann cells were quantified. Mann–Whitney test used for comparisons.

### 2.4 Generation of Nerves Partially Covered With Schwann Cells

Embryos were treated beginning at 22–23 hpf with 0.6 μM AG1478 (Cayman Chemical) in 1% DMSO (Chem‐Impex). Embryo water containing this ErbB inhibitor was changed daily. In different experiments, treatment with the inhibitor was either continued throughout injury and recovery or removed at the time of injury to allow nerve recovery in the absence of ErbB inhibitor. At 3 dpf, animals were mounted as above, the leading Schwann cell position was observed using epifluorescence microscopy, and the nerve was injured as above while choosing injury position to generate animals that were identically treated with the inhibitor but with either zero, one, 2–3, or many Schwann cells distal to the injury site. In these experiments, adjusting the injury site by 50–150 μm varied the number of distal Schwann cells from 0 to 2–3, but reduced the length of the proximal nerve segment only by 4% to 12%. Due to variability in Schwann cell migration or effect of ErbB inhibition, the leading Schwann cell position varied (Figure 6B). Animals with leading Schwann cells positioned at somite 10–16 were selected, allowing 14–20 somites of possible regrowth. After injury, full nerve transection and the number of Schwann cells distal to the injury site were assessed using confocal microscopy. Animals imaged on multiple days were individually housed in 6‐well plates.

### 2.5 Axonal Regrowth Rate Calculation

*NBT:dsRed* transgenic larvae were mounted, imaged, unmounted and imaged again at successive stages during development and axonal regrowth. Developmental growth rate was determined by measuring the entire length of the nerve at two different time points in the same individual and dividing the difference in length by the number of hours between imaging time points. Axonal regrowth rate was similarly determined; in some cases, the injury site was used as the landmark from which to assess the length of total regrowth. In the case of *erbb2* mutants, in which the site of injury was not easily determined at later stages because of axonal retraction and disrupted regrowth, the entire nerve was imaged so that the difference in length at the respective time points could be calculated. The rate in these cases was the difference in nerve length divided by the number of hours between the start of the two imaging sessions. Tiled images were assembled using the plugin MosaicJ (Thévenaz and Unser 2007) in Fiji/ImageJ (Schindelin et al. 2012).

### 2.6 Rate of Axonal Regrowth Before and After Contacting a Distal Schwann Cell Process

To quantify how axonal regrowth changed after Schwann cell contact, we analyzed one illustrative time lapse movie in which the time of initial contact was evident (Movie S1). Using the ImageJ line segment tool, we marked a spot slightly behind the growth cone in each frame and advanced frame by frame for the desired time window. Once the path length of regrowth was known, it was divided by the time interval. When the nerve was obstructed or out of frame at the precise time point, we adjusted the time to a point when the growth cone was again visible. Because the individual axons were sometimes thin and dim, it was necessary to greatly increase the saturation to visualize the most distal axon in the nerve at each timepoint.

### 2.7 In Situ Hybridization

Hybridization chain reaction (HCR) in situ hybridization was performed using the HCR Gold RNA‐FISH kit (Molecular Instruments) following the “Sample in solution” protocol, using probes (*ngfrb*, *erbb3b*, and *mGFP5*) and amplifiers at 1:50 (Molecular Instruments). Samples were probed at 37°C overnight and amplified at room temperature overnight. Prior to mounting in agarose, samples were washed in 5× SSC to eliminate detergent. Traditional in situ hybridization with digoxigenin‐labeled probes for *mfap4.1* was performed using a 628 bp probe (Zakrzewska et al. 2010), using standard protocols (Thisse and Thisse 2014). To enhance probe penetration in older zebrafish, larval samples were digested with proteinase K at 30 μg/mL for 23 min at 3 dpf or 36 min at 4 dpf.

### 2.8 Quantification and Statistical Analysis

Graphs and statistical analysis were performed in GraphPad Prism. Non‐normal distributions were compared using the Mann–Whitney test. Exact *p* values are reported.

## 3 Results

### 3.1 Mechanical Nerve Injury Model in Larval Zebrafish

Building on prior studies (Villegas et al. 2012; Moya‐Díaz et al. 2014) that employed electroablation and laser ablation of the larval zebrafish PLLN, we sought to develop a mechanical nerve transection model that was fast and reproducible with high recovery and survival rates. We employed an insect pin to transect the PLLN at 3 dpf (Figure 1A,D). A small incision was made at the location of the horizontal myoseptum of somite 7 of anesthetized larvae mounted in agarose. Soon after injury, confocal imaging was used to verify that the nerve was completely transected and that the spinal cord and other deep structures appeared to be intact. To observe neurons and Schwann cells, we used the transgenes *NBT:dsRed* and *foxd3:GFP*, respectively (Figure 1A) (Gilmour et al. 2002; Peri and Nüsslein‐Volhard 2008). The gap in the transected nerve typically spanned 50–100 μm (Figures 1 and S1). The injured area included superficial muscle and skin cells in addition to the nerve itself (Figure S1).

As in prior studies (Villegas et al. 2012; Ceci et al. 2014), the distal nerve segment was fragmented and being cleared by 5 h post transection (hpt) (Figure 1E). Around this same time, axons began to project from the proximal nerve segment through the injury site. We monitored axonal regrowth either by reference to the somite position or by length of axon from the injury site. The distal nerve segment through which axons regrew was a path of approximately 23 somites (1850 μm) at 3 dpf. Almost all wildtype animals showed significant axonal regrowth along the original path of the nerve by 24 hpt (Figures 1F, 10K, and S1) (average regrowth of 11 somites = 48% regrown, *n* = 20, 4 experiments) and complete regrowth by 48 hpt (Figures 1G,H and 10L) (full regrowth in 90% of animals, *n* = 21, 4 experiments). As noted in prior studies (Ceci et al. 2014), Schwann cells remained in both the proximal and distal nerve segments throughout regrowth, and the expression of the *foxd3:GFP* transgene was diminished specifically in the Schwann cells of the distal segment by 2 days post transection (dpt) (Figure 1G,H). This differential expression prompted us to further investigate other differences between proximal and distal Schwann cells.

### 3.2 Distal Schwann Cells Exhibit Characteristics of Repair Schwann Cells

Although the axons in almost all wildtype animals had completely regrown by 48 hpt (Figure 1), there was variability at the injury site, as evidenced both by the variable time proximal axons took to cross to the distal segment and by the number of misrouted proximal axonal branches crossing the injury site in different animals (Figures 2A–C and S1). Axonal misrouting (routing around the injured area) was apparent in 77% of injured animals (27 misrouted, *n* = 35 total, from 5 trials), while axons regrew along the horizontal myoseptum through the injury site in the remainder. Interestingly, the misrouted axonal branches frequently associated with Schwann cells strongly expressing *foxd3*:GFP (89%; 24 animals among 27 total with misrouting, 5 trials), whereas the axons regrowing along the proper path associated with distal Schwann cells with diminished *foxd3:GFP* (100% of injured animals, *n* = 35, 5 trials) (Figure 2A–C). Thus, these results suggested that the distal Schwann cells can guide regrowing axons along the proper path—a function reminiscent of repair Schwann cells in mammals (Son and Thompson 1995; Arthur‐Farraj et al. 2012; Cattin et al. 2015). As observed for mammalian repair Schwann cells (Arthur‐Farraj et al. 2012, 2017; Brosius Lutz et al. 2022), distal Schwann cells down‐regulate the myelination reporter *myelin basic protein:GFP* (*mbp:GFP*); expression was diminished at 1 dpt and 2 dpt, but was returning by 3 dpt (Figure 2D). After electroablation of the PLLN, Ceci et al. (2014) showed that expression of Mbp protein was reduced at 48 h post injury. Although both the *mbp:GFP* and *foxd3:GFP* transgenes demonstrate changes in expression in presumptive repair Schwann cells in the distal segment of the PLLN, these reporters follow different time courses. The down regulation of *foxd3:GFP* was only apparent at 2 dpt, and it continued to decrease at 3 dpt, when *mbp:GFP* was reactivated as the regrown axons became remyelinated by distal Schwann cells.

Mammalian repair Schwann cells are much longer than immature and myelinating Schwann cells (Gomez‐Sanchez et al. 2017), so we sought to measure the length of presumptive repair Schwann cells in the distal PLLN after injury. After PLLN outgrowth is complete, the number and density of Schwann cells in the nerve impede visualization of individual cells using *foxd3:GFP* (Figure 1) (Gilmour et al. 2002; Ceci et al. 2014). We found that individual Schwann cells can be visualized after injury at an earlier timepoint (30 hpf), so we measured the length of the Schwann cell migrating at the tip of the growing PLLN (Figure 2E–G), soon after nerve transection and 22 h later (Figure 2E′–G′). By this later timepoint, distal axons had degenerated and axonal regrowth had begun. By 22 hpt, distal Schwann cells had lengthened significantly (Figure 2E–H). These results indicate that at 1–2 dpt distal Schwann cells greatly elongate, downregulate myelin genes, and guide regrowing axons—all key features of repair Schwann cells as described in mammals. Furthermore, our results suggested that there may be functional differences in the ability of Schwann cells to guide regrowing axons because Schwann cells with low GFP expression associate specifically with properly routed axons, whereas Schwann cells with high GFP expression often associated with misrouted axons (Figure 2A–C).

To further compare distal Schwann cells in zebrafish with repair cells in mammals, we examined expression of the zebrafish orthologs of mammalian genes enriched in repair Schwann cells. *Ngfr* and *ErbB3* are expressed by developing Schwann cells, and previous transcriptomic analyses reported that both genes become upregulated in rodent repair Schwann cells, especially *Ngfr* (Taniuchi et al. 1988; Carroll et al. 1997; Brosius Lutz et al. 2022; Lovatt et al. 2022). Mammalian *Ngfr* has two zebrafish orthologs, *ngfra* and *ngfrb*. Expression of *ngfrb* increased in distal Schwann cells at 10 hpt, reached a peak at 1 dpt, then began to diminish by 2 dpt (Figure 3), when most axons had fully regrown in our injury model (Figure 1). At 24 hpt, there was also frequently upregulation of *ngfrb* in 1 or 2 proximal Schwann cells adjoining the injury site (Figure 3C; 86% of animals, *n* = 14). We found that *ngfrb* was uniformly upregulated in Schwann cells throughout the entire distal segment of the injured nerve at 24 hpt (Figure 3F). *erbb3b* was expressed at lower levels overall than *ngfrb*, but it was also differentially upregulated in distal Schwann cells at 24 hpt (Figure S2). These results provide additional evidence that distal Schwann cells in our larval zebrafish nerve injury model share many defining features of mammalian repair Schwann cells.

**FIGURE 3:**
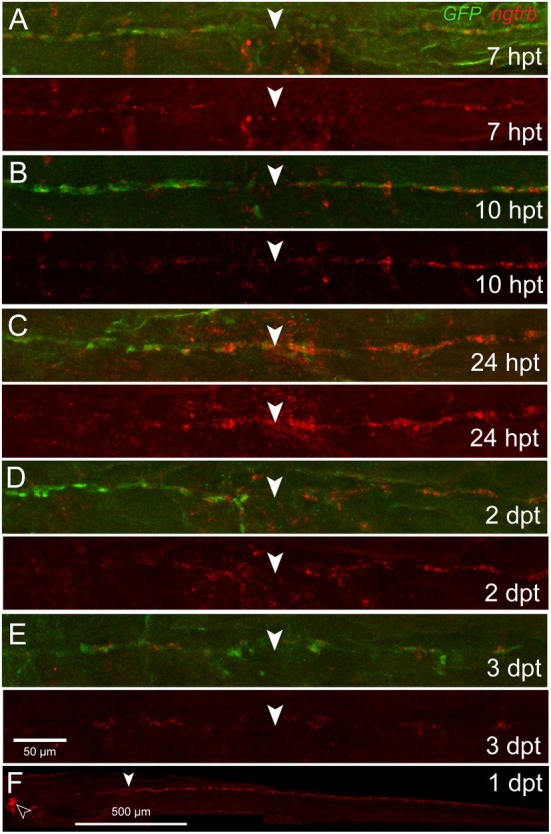
Distal Schwann cells increase *ngfrb* expression after nerve injury. Time course of *ngfrb* expression in wildtype larvae after injury at 3 dpf. (A–E) Endogenous *ngfrb* expression (red) and *foxd3:mGFP5* expression (green) were both detected in fixed animals by HCR in situ hybridization at the stages indicated. Top panel, merged image; bottom panel, *ngfrb* only. By 24 hpt, *ngfrb* was strongly expressed in cells distal to the injury site (arrowhead, distal segment to the right of the image), and 1 or 2 cells proximal to the injury site. By 3 dpt *ngfrb* expression was barely detectable. (F) Tiled image showing uniform and strong *ngfrb* expression along the length of the distal nerve segment, weak *ngfrb* signal in the proximal nerve segment, and strong expression in the pLLn ganglion (open arrowhead). We examined *ngfrb* expression after injury in 16 different trials at 10 different stages: 7 hpt (2 trials); 9 hpt (1 trial); 10 hpt (1 trial); 11 hpt (1 trial); 13 hpt (1 trial); 15 hpt (1 trial); 20 hpt (1 trial); 24 hpt (4 trials); 2 dpt (2 trials); and 3 dpt (2 trials). In each trial, 2–8 larvae (mean = 4) were mounted and examined by confocal microscopy. In all animals examined at all stages from 9 hpt to 2 dpt, distal Schwann cells upregulated *ngfrb* expression. Representative images are shown.

### 3.3 Axonal Regrowth Is Greatly Delayed in Mutants With Only a Few Schwann Cells

Previous analyses of laser ablation and electroablation of motor nerves and PLLN in zebrafish have reported that injured axons can regrow along aberrant paths in mutants lacking Schwann cells, which contrasts with conclusions in mouse indicating that Schwann cells have an important role in promoting axonal regrowth (Arthur‐Farraj et al. 2012; Villegas et al. 2012; Ceci et al. 2014; Rosenberg et al. 2014; Xiao et al. 2015; Jessen and Mirsky 2022; Bosch‐Queralt et al. 2023). To address this point in our mechanical injury model, we analyzed *erbb2* mutants, in which only a few Schwann cells are present (Figure 4) (Lyons et al. 2005). ErbB2 and its homolog ErbB3 together form a heteromeric receptor on Schwann cells that is activated by Neuregulin signals, and zebrafish *erbb2* mutants have disruptions in Schwann cells, other neural crest derivatives, and heart trabeculation (Lyons et al. 2005; Honjo et al. 2008; Liu et al. 2010). As in previous studies (Gilmour et al. 2002; Lyons et al. 2005), developmental outgrowth of PLLN axons appeared normal in *erbb2* mutants despite the strong reduction of Schwann cells (Figures 4B,C and 5). In contrast to wild‐type, in *erbb2* mutants both the proximal and distal segments of the injured nerve retracted away from the site of injury (Figure 4D; approximately 100 μm retraction in first hour after injury). Axonal regrowth was greatly delayed in *erbb2* mutants (Figures 4F,G, 5, and S3). Even by 2 dpt, when axons had fully regrown in almost all wild‐type animals (~23 somites), axons in *erbb2* mutants typically had regrown only 1 or 2 somites past the injury site (Figure S3C–E), and the rate of regrowth was approximately one‐sixth of wild‐type (Figure 5). Although Schwann cells can engulf degenerating axons (Catenaccio et al. 2017; Vaquié et al. 2019), we found that the distal axon was extensively fragmented by 5 hpt in *erbb2* mutants, a timeline comparable to wild‐type (Figure 4E). These results indicate that axonal regrowth after mechanical injury is greatly delayed in mutants with only a few Schwann cells.

**FIGURE 4:**
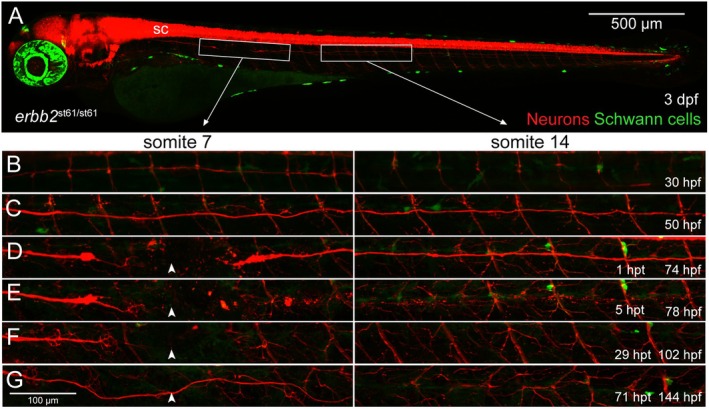
Time course of posterior lateral line development and recovery after transection in *erbb2* mutant. (A) Homozygous *erbb2* mutant larva with transgenic markers of Schwann cells (*foxd3:GFP*, green) and neurons (*NBT:dsRed*, red) one hour after mechanical injury of the PLLN at the region of somite 7 at 3 dpf. White boxes denote regions shown at higher magnification in subsequent panels. The spinal cord (sc) and other deep structures appeared intact. (B–G) Time course of images showing development and regrowth of PLLN after mechanical injury (arrowheads mark injury site). All images are from the same animal at the indicated stages. No Schwann cells were evident along the PLLN. In striking contrast to the wildtype animal (Figure 1), very few cells expressed the *foxd3:GFP* reporter in the mutant; two examples are apparent in D and E. The genotype was determined by PCR after imaging. Axonal regrowth in erbb2 mutants is quantified in Figure S3.

**FIGURE 5:**
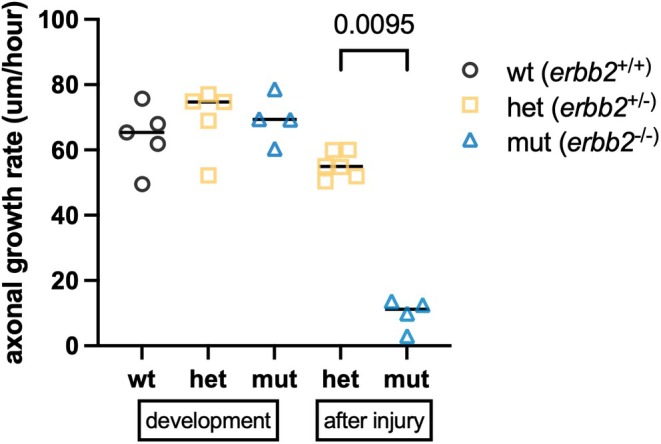
Rate of axonal outgrowth during development and regrowth after transection in wildtype and *erbb2* mutant larvae. Quantification of axonal growth rate in *erbb2* homozygotes compared to wildtype and heterozygous siblings. Rate of developmental outgrowth was calculated from 26 to 32 hpf, and the rate of regrowth was calculated from 1 to 2 dpt, after injury on 3 dpf. At the appropriate stages, axon lengths were measured in composite images of the PLLN. The genotypes of all animals were determined by PCR after imaging. Each point represents one nerve in one fish, measured at two timepoints to calculate the change in length over the intervening time. Lines indicate the median. Mann–Whitney test was used for comparisons.

### 3.4 A Single Distal Schwann Cell Can Promote Extensive Axonal Regrowth

Distal to the injury site, repair Schwann cells may support axonal regrowth by providing both stimulatory signals and a permissive substrate (Son and Thompson 1995; Yan et al. 1995; Parrinello et al. 2010; Rosenberg et al. 2014; Isaacman‐Beck et al. 2015). These repair Schwann cells could mediate axonal regrowth mainly by facilitating regrowth across the injury site, or they may function along the entire distal nerve segment. To determine where repair cells function during axonal regrowth, we used ErbB inhibitors to generate animals with PLLN axons only partly covered with Schwann cells. Pharmacological inhibition of ErbB signaling decouples axonal growth from Schwann cell migration (Lyons et al. 2005), and addition of ErbB inhibitor after nerve outgrowth has commenced results in nerves partially populated with Schwann cells (Figure 6A). We measured the position of the most distal Schwann cell in individual ErbB‐inhibited larvae (Figure 6B), and chose the injury site so that zero, one, a few, or many Schwann cells were located on the distal side of the transected nerve. Although the application of ErbB inhibitor may have systemic effects, by slightly varying the site of injury this experimental design allows comparison of animals which were identically treated with the ErbB inhibitor but which have differing numbers of distal Schwann cells. In all these conditions, proximal Schwann cells were present, and the length of the proximal nerve segment was minimally altered: varying the injury site position by a single Schwann cell length altered the proximal nerve segment length by less than 5% in an average animal (see Methods).

**FIGURE 6:**
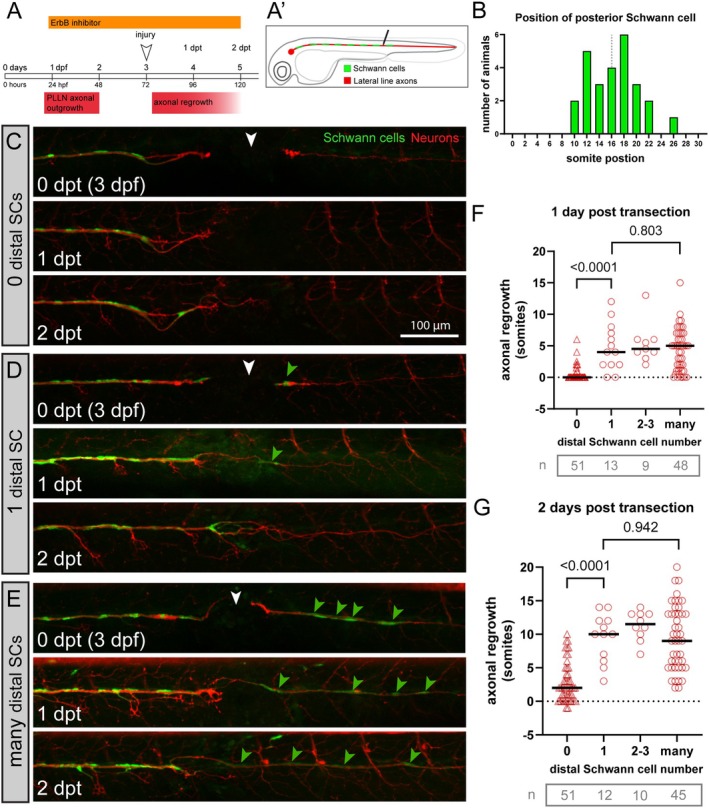
A single distal Schwann cell is sufficient for significant axonal regrowth after nerve transection. (A) Timeline of ErbB inhibitor treatment to generate nerves partially populated with Schwann cells. (A′) Transection position chosen to generate an injury site with a single distal Schwann cell. (B) Treatment with 0.6 μm AG1478 beginning at 23 hpf disrupts Schwann cell migration and results in a variable extent of Schwann cell position along the PLLN at 3 dpf. The Y axis represents the number of animals with a leading Schwann cell at the position noted on the X axis. Median position = somite 16 (dotted line), *n* = 26 animals. (C–E) Animals expressing *foxd3:GFP* (Schwann cells, green) and *NBT:dsRed* (neurons/axons, red) were longitudinally imaged from injury through 2 days post transection (white arrowhead at injury site, green arrowheads mark distal Schwann cells). (C) When the injury site was positioned to leave no distal Schwann cells, axons stall at the injury site 1 dpt. Only modest growth was observed at 2 dpt. (D) With a single distal SC, significant axonal growth was apparent at 1 dpt, and growth continued at 2 dpf. (E) When many distal Schwann cells were present, the extent of axonal regrowth was similar to (D). (F, G) Quantification of axonal regrowth at 1 dpt and 2 dpt, when 0, 1, 2–3, or many distal Schwann cells were present. Each point represents one nerve in one animal. Lines indicate the median. Data from four independent experiments, *n* = animals. Mann–Whitney test used for comparisons.

In animals with no distal Schwann cells, transected axons typically stalled at the injury site at 1 dpt; and they made little progress by 2 dpt (Figure 6C,F,G); these results are very similar to *erbb2* mutants (Figures 4 and S3). Strikingly, the presence of even a single distal Schwann cell promoted extensive axonal regrowth at 1 dpt and 2 dpt (Figure 6D,F,G). Animals with a few or many Schwann cells also showed extensive axonal regrowth, but the regrowth was not significantly better than animals with a single distal Schwann cell (Figure 6E–G). To test regrowth with intact ErbB signaling post injury, this experiment was repeated with the variation of washing out the ErbB inhibitor after injury (Figure S4). Again, in animals in which one distal Schwann cell was present, significantly more regrowth occurred than in animals with no distal Schwann cells (Figure S4C,D,F,G). The release of ErbB inhibition allows Schwann cells to resume migration (Figure S4B,D,E), in contrast to the case of continuous ErbB inhibition (Figure 6D,E). We sought to measure Schwann cell length as one indicator of repair Schwann cell state in these experiments, which was possible because Schwann cells were isolated distal to the injury site. We found that distal Schwann cells were significantly lengthened by 1 dpt (Figure 7A–D,G). Interestingly, this lengthening was sustained through 2 dpt only if ErbB inhibition was continuous (Figure 7E–G). These experiments indicated that repair Schwann cells have an especially important function immediately distal to the injury site. These analyses also indicate that transected axons that were able to navigate the injury site could subsequently regrow through large regions that were devoid of Schwann cells.

**FIGURE 7:**
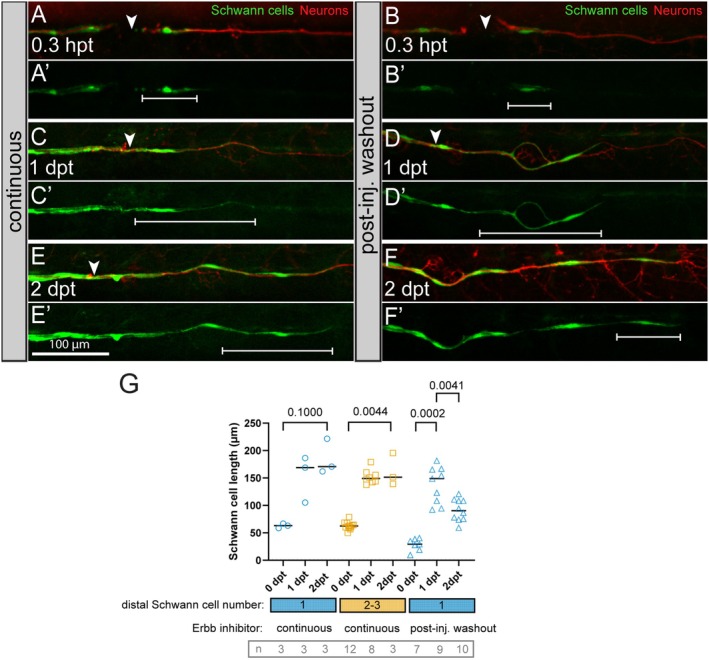
Individual distal Schwann cells lengthen after injury. All animals were treated with 0.6 μm AG1478 beginning at 23 hpf through 3 dpf, when an injury was positioned to generate 1 or a few distal Schwann cells (Figure 6A). (A–D) Schwann cells (*foxd3:GFP*, green) distal to the injury site lengthen by 1 day post transection (white arrowhead at injury site, white brackets mark distal Schwann cell length) as axons regrew (*NBT:dsRed*, red). (E) At 2 dpt, distal Schwann cells remained lengthened if ErbB signaling was inhibited throughout the experiment. (F) If ErbB inhibitor was removed at the time of injury, distal Schwann cell length began to decrease by 2 dpt, and significant Schwann cell migration was apparent, such that the injury site is no longer in the field of view. The same animals were imaged on successive days in these experiments. (G) Quantification of distal Schwann cell length. Only distal Schwann cells distinguishable from neighboring Schwann cells were quantified. Each point represents the length of one Schwann cell. Lines indicate the median. Data from three independent experiments, *n* = animals. Mann–Whitney test used for comparisons.

### 3.5 Contact With a Distal Schwann Cell Precedes Accelerated Axonal Regrowth Along the Correct Path

We sought to further investigate the interactions between regrowing axons and distal Schwann cells at the injury site using time‐lapse imaging (Figure 8). The figure depicts frames from a single illustrative time‐lapse movie that shows axons regrowing dorsally and ventrally from the injury site, beginning at the time when a distal Schwann cell process first contacts a ventral axon (Figure 8C, inset). Fifty minutes after this initial contact, a new axonal branch appeared at precisely the contact point (Movie S1). This new branch then followed the path formed by the distal Schwann cell process across the injury site and into the distal nerve segment, where it regrew along a path of distal Schwann cells (Figure 8D). Though this branch was formed later than many other branches that did not contact distal Schwann cells, it was the first to cross the injury site. After Schwann cell contact, the crossing axon appeared to accelerate as it regrew distally through the imaged region (Figure 8B,D). During the first 2 h after Schwann cell contact, the axon grew at a rate of 30 μm per hour. By 10 h after contact, the axon was regrowing at a rate similar to developmental outgrowth (compare Figure 8D to Figure 5), and more than double the growth rate of a misrouted axon that projected dorsally and was never contacted by a Schwann cell (Figure 8B). Analysis of this movie provides evidence that distal Schwann cell contact precedes axonal crossing of the injury site and accelerated regrowth along the correct path.

**FIGURE 8:**
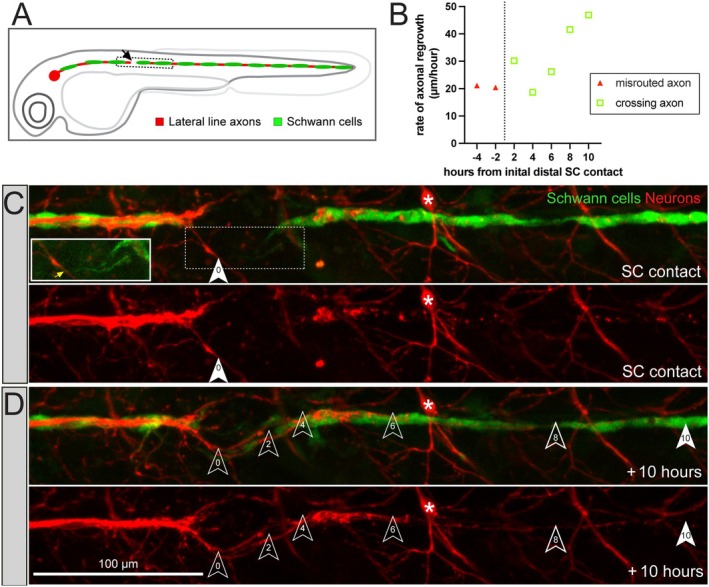
Time‐lapse analysis of interaction between distal Schwann cells and axons regrowing after transection. (A) Diagram of larval fish with injury and imaging sites labeled. A wildtype larva with the Schwann cell and neuronal reporters was injured at 3 dpf and analyzed with time‐lapse imaging from 8 to 34 hpt. The results in this figure derive from the analysis of a single illustrative time‐lapse movie. The initial point at which a distal Schwann cell process contacted a regrowing axon was determined, allowing analysis of axons before and after contact. (B) Quantification of growth rate of an early, mistargeted axon bundle which did not contact a distal Schwann and grew on an aberrant path (red triangles) and the axon bundle that was first contacted by a distal Schwann cell (green squares). The rate of axonal regrowth accelerated at 8 h after Schwann cell contact. (C) Frame from the timelapse movie at the point when a distal Schwann cell first contacted a regrowing axon (arrowhead marked 0). Inset depicts brightened region denoted by dotted box, and yellow arrow marks Schwann cell‐axon contact point. (D) Frame from the movie 10 h after contact, showing that a new axon branch grew from the point of Schwann cell contact across the injury site and down the path of the PLLN. Arrowheads indicate the position of the most distal axon at 2‐h intervals after Schwann cell contact as indicated by the numbers in each arrowhead. Asterisk marks a motor neuron that is evident throughout the analysis.

### 3.6 Axonal Regrowth Occurs Despite Strong Reduction of Macrophages

Schwann cells recruit macrophages to the injured nerve, where they secrete cytokines and help phagocytose myelin and axonal debris (Perry and Brown 1992; Liu et al. 1995; Shamash et al. 2002). In addition to debris clearance, there is evidence that macrophage‐endothelial cell communication can establish a cell bridge that guides regrowing axons of transected nerves (Cattin et al. 2015; Li et al. 2022). To examine the role of macrophages in our mechanical nerve injury model, we employed *irf8* mutants, which have only a few macrophages at the stages we examined (Shiau et al. 2015). We visualized the macrophage response with the *mpeg1:GFP* transgene, which is expressed mainly in the myeloid lineage in the larva (Ellett et al. 2011), but also labels a few other immune cell types at lower levels (Lin et al. 2019; Ferrero et al. 2020; Zhao et al. 2023).

To examine the macrophage response to nerve injury, we imaged *mpeg1:GFP* and *NBT:dsRed* throughout the entire animal, starting soon after injury and continuing to 2 dpt (Figure 9). In heterozygous siblings, many macrophages, evident from their characteristic branched morphology and strong expression of *mpeg1:GFP*, were present in the region of the PLLN soon after injury, whereas only a few macrophages were evident in *irf8* mutants (Figure 9A,B; a single bright green cell without complex morphology is evident in Figure 9B). In wildtype siblings, macrophages from across the body concentrated at the injury site by 6 hpt (Figure 9A′). In *irf8* mutants, no macrophages highly expressing *mpeg1:GFP* were apparent, although some weakly expressing cells were visible at the injury site (Figure 9B′). The cells weakly expressing *mpeg1:GFP* in *irf8* mutants may be metaphocytes, which express this transgene and begin to appear in the skin at about 4 dpf (Lin et al. 2019, 2020; Zhao et al. 2023). To address the identity of these cells, we examined the macrophage marker *mfap4.1* (Zakrzewska et al. 2010) in wildtype and *irf8* mutant siblings within the first day post transection (Figure S5). Expression of *mfap4.1* was apparent at the injury site in wildtype animals at 6 hpt and 1 dpt, but no *mfap4.1* signal was detected in *irf8* mutants (Figure S5), as shown previously (Shiau et al. 2015; Tsarouchas et al. 2018). These results indicate that there is an extensive macrophage response at the injury site within 6 hpt, and that there is a very strong reduction of macrophages in *irf8* mutants at the stages we analyzed, consistent with numerous prior studies (Shiau et al. 2015; Tsarouchas et al. 2018; Zhao et al. 2018).

**FIGURE 9:**
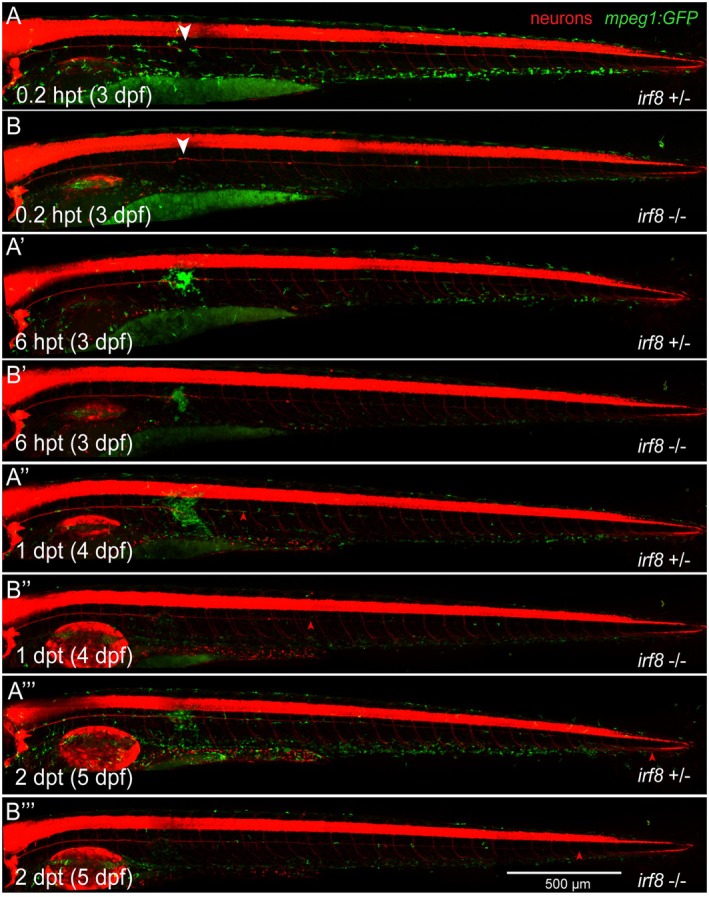
Macrophage numbers and injury response in wildtype and *irf8* mutant animals. (A, B) Time course of heterozygous and homozygous mutant *irf8* siblings expressing *NBT:dsRed* (neurons, red) and *mpeg1:GFP* (myeloid cells, green). The *mpeg1:GFP* transgene is expressed highly in macrophages, which have characteristic complex branched morphologies. Numerous macrophages are apparent in heterozygous animals, while *irf8* mutants have few, if any macrophages at 3 dpf. White arrowheads = injury site. (A′, B′) 6 h post transection, macrophages have migrated from around the body to concentrate at the injury site. In *irf8* mutants, green *mpeg1:GFP* signal was apparent at the injury site, but cells do not have branched morphology characteristic of macrophages. Distal axons fragmented normally in *irf8* mutants. (A″, B″) In heterozygous animals at 1 dpt, macrophages became less dense at the injury site and occupied the injured muscle unit (somite), localizing in the characteristic chevron shape of a somite. In *irf8* mutants at 1 dpt, only a few macrophages were present, and cells weakly expressing the *mpeg1:GFP* transgene are no longer apparent at the injury site. Axonal debris has been cleared and axonal regrowth is occurring similarly in both heterozygous and mutant animals (red arrowheads = axonal regrowth extent). (A‴, B‴) By 2 dpt, macrophages began to leave the injury site in heterozygous animals. Almost complete axonal regrowth was observed in this *irf8* mutant at 2 dpt (see also quantitation in Figure 10). Several macrophages with branched morphology are visible in the *irf8* mutant by 5 dpf. Genotypes were determined by PCR after imaging.

Despite the strong reduction of macrophages in *irf8* mutants, the distal segment of the injured nerve was fragmented and cleared similarly in wildtype and *irf8* mutant siblings (Figure 10C,D,I,J). Axonal regrowth was indistinguishable in *irf8* mutants and wildtype siblings at 1 dpt and 2 dpt (Figure 10E–H,K,L). In summary, our analysis of *irf8* mutants indicates that axonal degradation, clearance, and regrowth can occur despite the presence of only a few macrophages in these animals.

**FIGURE 10:**
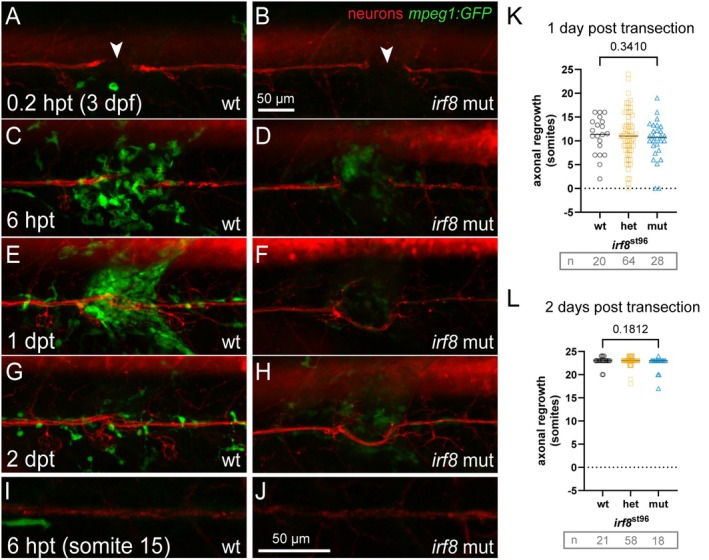
Normal axonal regrowth in *irf8* mutants. (A–J) Time course of wild type and homozygous mutant *irf8* siblings expressing *NBT:dsRed* (neurons) and *mpeg1:GFP* (myeloid cells). The PLLN was injured at 3 dpf, and the time points of the images are indicated. (A, C, E, G) In a wild type animal imaged at the injury site at different stages, many macrophages were apparent and the distal axons were fragmented at 6 hpt. By 2 dpt, fewer macrophages were present and the axons were regrown. (B, D, F, H) In the *irf8* mutant sibling, very few weakly GFP‐expressing cells were evident. Axon regrowth occurred on the same timescale as in the control sibling. (I, J) Images from the same wildtype and *irf8* mutant siblings showing axonal debris distal to the injury site. Genotypes were determined by PCR after imaging. (K, L) Quantification of axonal regrowth at 1 dpt and 2 dpt in the indicated genotypes. Each point represents one nerve in one animal. Lines indicate the median. Data from four independent experiments, *n* = animals. Mann–Whitney test used for comparisons.

## 4 Discussion

Building on previous studies of nerve repair in zebrafish, our analysis indicates that mechanical nerve injury triggers the formation of repair Schwann cells that display many characteristics previously defined in their mammalian counterparts. In adult mammals, nerve injury activates the formation of repair Schwann cells that perform several key activities, including down‐regulation of myelin genes and lengthening to form a path for regrowing axons (Jessen and Mirsky 2016). Injured axons regrow much more rapidly in zebrafish larvae than in adult rodents, but our analysis together with prior studies indicates that many features of repair Schwann cells are nonetheless conserved between the two systems.

Mechanical transection of the nerve leads to rapid fragmentation of the distal axonal segment, which occurs in about 5 h in the zebrafish larval PLLN (Figure 1E) (Villegas et al. 2012; Ceci et al. 2014). Schwann cells begin to extend processes into the injury site by 6 hpt (Figure 8) (Ceci et al. 2014; Xiao et al. 2015). By 10 hpt, distal Schwann cells have upregulated expression of *ngfrb*, the zebrafish ortholog of the well‐known mammalian repair Schwann cell gene *Ngfr* (Figure 3) (Taniuchi et al. 1988). Expression of *ngfrb* and another gene upregulated in repair cells, *erbb3b*, reached a peak in distal Schwann cells at about 1 dpt (Figures 3 and S2) (Carroll et al. 1997; Lovatt et al. 2022). Previous studies in mammals reported that p‐ERK increased in repair cells at different positions distal to the injury site, and also in Schwann cells immediately proximal to the injury site (Sheu et al. 2000; Harrisingh et al. 2004). Similarly, our analysis showed that *ngfrb* expression increased along the entire length of the distal nerve segment, and in one or two cells just proximal to the injury site (Figure 3C,F). By 1 day post transection, distal Schwann cells doubled their length (Figure 2E–H) and lost expression of the *mbp:GFP* reporter and Myelin Basic Protein (Figure 2D) (Ceci et al. 2014). At this time, there is significant axonal regrowth across the injury site in almost all wildtype animals, and properly routed axons are growing in close association with distal repair cells, which can be distinguished by persistent changes in *foxd3:GFP* reporter expression and upregulation of *ngfrb* and *erbb3b* (Figures 2, 3, and S2). In wildtype larvae 3 days after transection, the axons were fully regrown and remyelination was well underway (Figures 1 and 2) (Ceci et al. 2014). Thus, nerve injury triggers a similar sequence of responses in larval zebrafish and adult mammals (Coleman and Freeman 2010; Villegas et al. 2012; Gomez‐Sanchez et al. 2017; Brosius Lutz et al. 2022). The combined results of these studies show that the key features of repair Schwann cells are conserved in larval zebrafish and adult mammals.

Our analysis of the zebrafish larval system supports two key conclusions about the functions of Schwann cells at different positions within the regrowing nerve. First, proximal Schwann cells are not sufficient to direct regrowing axons across the injury site. By analyzing animals in which Schwann cells populated only the proximal nerve segment, we found that axonal regrowth failed after a distal injury (Figure 6C,F,G). These experiments involved systemic application of ErbB inhibitor, which may affect the state of any cell in the animal. Nonetheless, in animals identically treated with the ErbB inhibitor but with a single Schwann cell distal to the injury, axonal regrowth succeeded (Figure 6D,F,G). In addition, we repeated the experiments with removal of ErbB inhibitor at the time of injury, and the results similarly illustrated the importance of distal Schwann cells for axonal regrowth (Figure S4). Thus, in our experimental model, proximal Schwann cells alone are not sufficient for axonal regrowth. In contrast, there is evidence in mammals that proximal Schwann cells may lead axonal branches regrowing through the injury site from the proximal nerve stump (Cattin et al. 2015). The nerve gap is 15–30 times longer in adult mice than larval zebrafish (Cattin et al. 2015) (Figure 1), and it is likely that different repair mechanisms are required to bridge these injuries. In the rodent model, blood vessels first grow across the gap, and it is possible that these allow proximal Schwann cells to migrate across the large injury site and guide regrowing axons. In any case, our results emphasize the essential role of distal Schwann cells in guiding and stimulating axonal regrowth after mechanical injury (Figures 6D,E and 8).

Second, a single distal repair Schwann cell can direct regrowing proximal axons across the injury site and allow for their extensive subsequent regrowth. The key evidence supporting this conclusion derives from analysis of nerves partially populated with Schwann cells, in which the injury is positioned so that only one Schwann cell is distal to the point of transection (Figure 6D,F,G). In these cases, proximal axons can cross the injury site and grow posteriorly over long distances that are not and have never been populated by Schwann cells. Timelapse observation also emphasizes the activity of Schwann cells immediately distal to the injury site: soon after distal Schwann cell contact, an extending proximal axon redirected toward the correct path and subsequently accelerated growth (Figure 8). Our conclusion about the necessity of distal Schwann cells contrasts with a study that addressed the role of distal Schwann cells in 1 month old zebrafish (Graciarena et al. 2014). Those authors laser‐ablated cells immediately distal to the nerve cut and found that axons can regrow through the site in which distal Schwann cells had been ablated. It is possible that the laser ablation did not completely remove substrates or guidance cues that were produced by distal Schwann cells before they were ablated.

Our analyses show that axons can regrow through regions devoid of Schwann cells after they successfully navigate the injury site (Figure 6D,F,G). This parallels the ability of axons to extend along the correct path during their developmental outgrowth in *erbb2* mutants, which have only a few Schwann cells (Figure 4B,C) (Gilmour et al. 2002; Lyons et al. 2005; Perlin et al. 2011). Because our model involves injury less than 2 days after initial nerve outgrowth, it is possible that developmental cues persist and guide the regrowing PLLN axons; further work on the guidance mechanisms is required to address this point. Our results also appear to contrast with the prevailing view from mammalian studies that repair Schwann cells and the Büngner bands they form are required along the entire length of the distal nerve segment (Jessen and Mirsky 2016). This seeming discrepancy may reflect the different scales of the two systems—the zebrafish PLLN at 6 dpf is roughly 10 times shorter than the adult mouse sciatic nerve—and also the possible persistence of developmental cues mentioned above.

Important questions remain outstanding about the nature of the signals that instruct Schwann cells to enter and exit the repair state. Our results support a model in which axonal contact maintains the myelinating Schwann cell state, via a currently unknown signal. In the absence of this axonal signal, Schwann cells rapidly transition to the repair state, such that Schwann cells begin to extend fine processes and transcribe repair cell genes within a few hours of axonal fragmentation (Figures 1, 3, and 8). The length of repair cells and expression of repair cell genes both peak by 24 hpt, when axons have regrown significantly in wildtype larvae (Figures 1, 2, 3, and 7). By 2 dpt, axonal regrowth is largely complete, Schwann cell length and expression of repair genes have diminished, and *mbp* expression is again apparent in distal Schwann cells, as remyelination proceeds. Our results provide evidence that ErbB signaling is required for Schwann cells to exit the repair state (Figure 7). In the presence of ErbB inhibitor, distal Schwann cells reestablished axonal contact but remained elongated at 2 dpt, whereas Schwann cells were significantly shorter in control animals with restored ErbB signaling (Figure 7E–G). Similarly, previous studies in mammals show that axonal Nrg1 signals are required for robust remyelination of axons that had regrown after injury (Fricker et al. 2011, 2013). The addition of ErbB inhibitor alone did not cause Schwann cells to lengthen, and distal Schwann cells had normal length shortly after injury in ErbB‐inhibited animals (Figure 7A). This indicates that loss of ErbB signaling alone does not cause Schwann cells to enter the repair state. Similarly, prior evidence in mammals shows that mature Schwann cells maintain the myelinating state (i.e., do not become repair cells) after loss of ErbB2 function (Atanasoski et al. 2006). In summary, our results together with prior studies suggest that Nrg1 signals from regrowing axons are required for Schwann cells to exit the repair state and that loss of a distinct, unknown axonal signal instructs Schwann cells throughout the distal nerve segment to enter the repair state after axonal fragmentation.

### 4.1 Role of Macrophages in Axonal Regrowth

By analyzing *irf8* mutants in our larval PLLN injury model, we found that, surprisingly, macrophages were not required for debris clearance or axonal regrowth (Figures 9 and 10). Prior studies in zebrafish and mammals show that macrophages phagocytose axonal debris after injury (Stoll et al. 1989; Rosenberg et al. 2012), but the requirement for macrophages is less clear. For example, one study of larval zebrafish nerve injury showed that depletion of macrophages only slowed debris clearance by a few hours (Villegas et al. 2012), and another reported that epidermal cells clear sensory axonal debris with no requirement for Schwann cells or macrophages (Rasmussen et al. 2015). Moreover, in mouse models with depleted macrophages, myelin debris clearance was only slightly changed (Perry et al. 1995), and axonal regrowth was somewhat diminished (Dailey et al. 1998). Although we found no requirement for macrophages in axonal regrowth of the larval PLLN, it remains important to test the possible roles of macrophages in specific nerve injury contexts. For example, it has been proposed that macrophages are important to bridge large gaps in transected mammalian nerves (Cattin et al. 2015), which may not be necessary with the more limited lesions that we have analyzed.

### 4.2 Limitations

Our analysis focused entirely on mechanical injury of the PLLN in early larval zebrafish. Comparisons among vertebrate models provide evidence that the functions of repair Schwann cells are conserved, but there may be important differences in the role of repair Schwann cells and other cell types in different nerves, injury types, stages, or species. For example, developmental axon guidance cues may still be operative and involved in axonal regrowth at the stages we analyzed, and the path length of axonal regrowth in our injury model is significantly shorter than in mammalian models and clinical settings. Identification of the pathways controlling the formation and function of repair Schwann cells will be required to determine if there are important differences in repair Schwann cells operating in different circumstances or species. In addition, we did not assess functional recovery or long‐term maintenance of the lateral line system, so repair Schwann cells and other cell types may have important roles in nerve repair that were not evident from our transgenic reporter analysis.

## Funding

This work was supported by the National Institutes of Health/NINDS (R35 NS111584 to W.S.T.).

## Conflicts of Interest

The authors declare no conflicts of interest.

## Supporting information


**Figure S1:** Extent of the mechanical injury site and axonal regrowth at 1 day post transection. (A) Full body tiled image of a wildtype larva with Schwann cells (green) and neurons (red) labeled. Spinal cord, SC. (B) Corresponding transmitted PMT image with the injury site denoted by an open arrowhead. (C–F) Merged images depicting neurons (red) and tissue surround the injury site (gray). (C′–F′) Corresponding transmitted PMT images.

**Figure S2:** Distal Schwann cells express *erbb3b* after injury. Time course of *erbb3b* expression in wildtype larvae after injury at 3 dpf. Endogenous *erbb3b* expression in red; *foxd3:mGFP5* expression in green, both detected by HCR in situ hybridization. (A and B) Top panel: merged image, bottom panel: *erbb3b* only. (A) At 7 hpt, low levels of *erbb3b* expression are apparent along the pLLn, with similar expression proximal and distal to the injury site (white arrowheads). (B) By 24 hpt, *erbb3b* expression is differentially upregulated in Schwann cells in the distal nerve segment. We examined *erbb3b* expression after injury in 2 trials at 7 hpt and 3 trials at 24 hpt. In each trial, 2–8 larvae (mean = 3.4) were mounted and examined by confocal microscopy. Representative images are shown.

**Figure S3:** Axonal regrowth in *erbb2* mutants. (A) Diagram depicting zebrafish larval somite numbering scheme, location of posterior lateral line nerve injury, and axonal regrowth measurement paradigm. Axonal regrowth was measured as the number of somites (muscle segments) through which the most distal axon has grown. (B) Axonal regrowth at 1 day post transection (dpt) in *erbb2* mutants was significantly lower than in wild type or heterozygous siblings. In *erbb2* mutants, axonal retraction after injury was observed frequently, resulting in negative regrowth. Each point represents one nerve in one animal. (C–E) Injury site (white arrowhead) images showing axonal regrowth paths (dotted line) at 2 dpt. Axons (*NBT:dsRed*, red) in wild type and heterozygous animals route around the injury site and reacquire the normal nerve path, while in *erbb2* mutants, axonal regrowth was minimal, even after 2 days. (C′–E′) Wild type and heterozygous animals had Schwann cells expressing the *foxd3:GFP* transgene (green), whereas no Schwann cells expressing this reporter were evident in *erbb2* mutants. All animals were genotyped by PCR after imaging, *n* = animals. Mann–Whitney test was used for comparisons.

**Figure S4:** After ErbB inhibitor washout, a single Schwann cell was sufficient for significant axonal regrowth after nerve transection. (A) Timeline of ErbB inhibitor treatment to generate nerves partially covered in Schwann cells. ErbB inhibitor is removed post injury. (A') Transection paradigm to generate an injury site with a single distal Schwann cell. (B) While initial Schwann cell migration was disrupted by ErbB inhibition, after injury and removal of ErbB inhibitor, Schwann cells migrated an average of ~5 somites further posterior over 2 days. The Y axis represents the number of animals with a leading Schwann cell at the position noted on the X axis. Median positions indicated by dotted lines; at 3 dpf Mdn = 7; at 5 dpf Mdn = 11.5; the same 34 animals were assessed at both timepoints. (C–E) Animals expressing *foxd3:GFP* (Schwann cells, green) and *NBT:dsRed* (axons, red) were imaged at different time points from injury through 2 days of regrowth (white arrowhead at injury site, green arrowheads mark distal Schwann cells) (C) Without a distal Schwann cell, axons stalled at the injury site at 1 dpt. Only modest growth was observed at 2 dpt. (D) With a single distal Schwann cell, significant axonal growth was apparent at 1 dpt and continued at 2 dpt. (E) Without ErbB inhibitor and with many distal Schwann cells, axonal regrowth was slightly more than when one distal Schwann cell was present. Schwann cell migration resumed after ErbB inhibitor was removed, as illustrated by the more posterior positions of indicated distal Schwann cells. (F, G) Quantification of axonal regrowth at 1 dpt and 2 dpt. Each point represents one nerve in one animal. Data from three independent experiments, *n* = animals. Lines indicate the median. Mann–Whitney test used for comparisons.

**Figure S5:** Macrophage marker *mfap4.1* expression is not detected in *irf8* mutant animals. (A–F′) *mfap4.1* expression was detected by in situ hybridization in injured animals fixed at the indicated timepoints (black arrowheads = injury site). (A'–F′) Higher magnification images of the same animals as in (A–F). (A, A′, B, B′) At 3 dpf soon after injury, *mfap4.1* expression is detected in the head, body, and posterior blood island (open arrowheads) of wildtype animals while no *mfap4.1* expression is detected in *irf8* animals. (C, C′, E, E′) At 6 hpt, *mfap4.1* expression is apparent at the injury site in wildtype animals, increasing by 1 dpt. (D, D′, F, F′) *mfap4.1* expression is not detected at the injury site in *irf8* mutant animals. Animals were genotyped by PCR after injury, fixation, and imaging. Representative images from cohorts of animals (0.6 hpt: wt = 5, mut = 5; 6 hpt: wt = 5, mut = 2; 1 dpt: wt = 5, mut = 5).

**Movie S1:** In a wild type animal with labeled neurons (*NBT:dsRed*, red) and Schwann cells (*foxd3:GFP*, green), the PLLN was transected as described above at 3 dpf. This timelapse of the injury site begins 8 h post transection and runs for 25 h, with an interval of 10 min between frames. The distal Schwann cell at the injury site makes contact with a proximal axon projecting ventrally at 5.3 h into the movie (frame 32). After contact the axon proceeded along the distal Schwann cell process until it reached the main distal nerve segment, and regained the correct course. The axon analyzed has projected down the main trunk of the PLLN and passed out the field of view at 15 h (frame 92). Single axons are thin and dim in comparison to the intact nerve; fine processes that are not evident in the timelapse as shown can be resolved after brightness is adjusted (e.g., Figure 8C inset).

## Data Availability

The data that support the findings of this study are available from the corresponding author upon reasonable request.
